# Septic Cardiomyopathy

**DOI:** 10.31083/j.rcm2501023

**Published:** 2024-01-15

**Authors:** Ivana Lukić, Damir Mihić, Silvija Canecki Varžić, Kristina Selthofer Relatić, Lada Zibar, Domagoj Loinjak, Željka Breškić Ćurić, Lucija Klobučar, Lana Maričić

**Affiliations:** ^1^Faculty of Medicine, University J. J. Strossmayer in Osijek, 31000 Osijek, Croatia; ^2^Department of Heart and Vascular Diseases, University Hospital Centre Osijek, 31000 Osijek, Croatia; ^3^Department of Pulmology and Intensive Care Medicine, University Hospital Centre Osijek, 31000 Osijek, Croatia; ^4^Department of Endocrinology, University Hospital Centre Hospital Osijek, 31000 Osijek, Croatia; ^5^Department of Nephrology, University Hospital Merkur, Zagreb, 10000 Zagreb, Croatia; ^6^Department of Internal Medicine, General Hospital Vinkovci, 32100 Vinkovci, Croatia

**Keywords:** septic cardiomyopathy, sepsis-induced myocardial dysfunction, mitochondrial dysfunction, echocardiography, biomarker, fibroblast growth factor-21, growth differentiation factor-15

## Abstract

Sepsis is defined as a life-threatening organ dysfunction caused by a dysregulated host response to infection. Sepsis-induced myocardial dysfunction represents reversible myocardial dysfunction which ultimately results in left ventricular dilatation or both, with consequent loss of contractility. Studies on septic cardiomyopathy report a wide range of prevalence ranging from 10% to 70%. Myocardial damage occurs as a result of weakened myocardial circulation, direct myocardial depression, and mitochondrial dysfunction. Mitochondrial dysfunction is the leading problem in the development of septic cardiomyopathy and includes oxidative phosphorylation, production of reactive oxygen radicals, reprogramming of energy metabolism, and mitophagy. Echocardiography provides several possibilities for the diagnosis of septic cardiomyopathy. Systolic and diastolic dysfunction of left ventricular is present in 50–60% of patients with sepsis. Right ventricular dysfunction is present in 50–55% of cases, while isolated right ventricular dysfunction is present in 47% of cases. Left ventricle (LV) diastolic dysfunction is very common in septic shock, and it represents an early biomarker, it has prognostic significance. Right ventricular dysfunction associated with sepsis patients with worse early prognosis. Global longitudinal stress and magnetic resonance imaging (MRI) of the heart are sufficiently sensitive methods, but at the same time MRI of the heart is difficult to access in intensive care units, especially when dealing with critically ill patients. Previous research has identified two biomarkers as a result of the integrated mitochondrial response to stress, and these are fibroblast growth factor-21 (FGF-21) and growth differentiation factor-15 (GDF-15). Both of the mentioned biomarkers can be easily quantified in serum or plasma, but they are difficult to be specific in patients with multiple comorbidities. Mitochondrial dysfunction is also associated with reduced levels of miRNA (microRNA), some research showed significance of miRNA in sepsis-induced myocardial dysfunction, but further research is needed to determine the clinical significance of these molecules in septic cardiomyopathy. Therapeutic options in the treatment of septic cardiomyopathy are not specific, and include the optimization of hemodynamic parameters and the use of antibiotic thera-pies with targeted action. Future research aims to find mechanisms of targeted action on the initial mechanisms of the development of septic cardiomyopathy.

## 1. Introduction

Sepsis-induced myocardial dysfunction represents reversible myocardial dysfunction which ultimately results in left ventricular dilatation or both, with consequent loss of contractility. Sepsis is defined as a life-threatening organ dysfunction caused by a dysregulated host response to infection [1]. The current definition of sepsis emphasizes the presence of organ dysfunction. Cardiac dysfunction caused by inflammation and systemic redistribution of blood volume plays a key role in this but worsens with reduced tissue oxygen utilization [2]. According to the research that has been done so far, there are two basic mechanisms that lead to myocardial dysfunction in sepsis; on the one hand, it is a consequence of the direct action of the pathogen, and on the other hand, the activation of the host’s immune system [3, 4]. From a pathophysiological point of view, myocardial damage occurs as a result of weakened myocardial circulation, direct myocardial depression, and mitochondrial dysfunction [4]. All three mechanisms intertwine with each other at the same time. Although all three mechanisms are equally important, recent studies emphasize that mitochondrial dysfunction is the key factor in the development of cardiac dysfunction.

This review paper aims to present the mechanisms of the development of cardiac dysfunction of the myocardium, diagnostic methods, and potential therapeutic goals with an emphasis on mitochondrial dysfunction.

## 2. Septic Cardiomyopathy

In 1921, E. Romberg first described septic cardiomyopathy as “septic acute myocarditis” in his Textbook of Heart and Vascular Diseases [5]. In 1967, McLean *et al*., [6] by conducting a clinical trail, described diagnostic criteria for detecting heart failure as part of sepsis included a low cardiac index (CI). In 1984, Parker *et al*. [7] using radionuclide angiography, defined septic cardiomyopathy as reversible myocardial depression due to sepsis and septic shock, defined as a reduced left ventricular ejection fraction (LVEF <40%), and an increase in mean end-diastolic volume. Systolic blood pressure (end-diastolic volume (EDV), end-systolic volume (ESV)), excluding acute coronary events. This usually occurs within 2 to 3 days after the onset of sepsis and resolves within 7 to 10 days. Studies of septic cardiomyopathy report prevalence rates ranging from 10% to 70%. A 2023 meta-analysis database (Cochrane Central Register of Controlled Trials, MEDLINE, and Embase) found a prevalence of septic cardiomyopathy of 20% in patients with sepsis [8]. The epidemiology of septic cardiomyopathy remains unclear as there is no consensus on the definition [9]. According to the available research, the risk factors that stand out are male gender, age, high lactate levels at admission, and pre-existing heart diseases. Based on current knowledge, septic cardiomyopathy is described by echocardiographic findings as: ventricular dilation with increased ventricular compliance and normal to low filling pressures, in contrast to the pattern of cardiogenic shock where ventricular pressures are elevated. There is a reduced LVEF, without a decrease in stroke volume (SV) and recovery of function within 7–10 days. Diminished response to volume replacement and administration of catecholamines. It can be isolated systolic or diastolic dysfunction of the left ventricle, and both or only the right ventricle can be affected. Cardiac magnetic resonance imaging (Cardiac MRI) shows changes suggestive of myocardial edema or altered metabolic status, a pattern distinct from that of ischemia and necrosis. Some theories describe septic cardiomyopathy as a protective state of “hibernation” of the myocardium [10]. Based on clinical, echocardiographic, and biochemical parameters, Geri *et al*. [11] presented five different hemodynamic phenotypes of septic cardiomyopathy: (1) absence of cardiac dysfunction; (2) left ventricular systolic dysfunction; (3) hyperkinetic profile, preserved or supernormal left ventricular systolic function with elevated aortic blood flow velocities; (4) right ventricular (RV) failure; (5) persistent hypovolemia. In the aforementioned research, cluster analysis based on five different cardiovascular phenotypes showed that 16.9% belonged to cluster 1 as “well resuscitated”, 17.7% had an “left ventricle (LV) systolic dysfunction” phenotype (cluster 2). 23.3% had a phenotype reflecting a “hyperkinetic” state (cluster 3), 22.5% had a hemodynamic profile consistent with “RV failure” (cluster 4) and to last cluster 5, “still hypovolemic” belonged to 19.4%. Seven-day mortality and mortality in the intensive care unit (ICU) was 20.1% and 35%, respectively. The highest mortality was recorded in cluster 4 (34% in ICU, and 7-day mortality was 22%) [11]. Previous research has not established a clear definition of septic cardiomyopathy, and the main reasons are insufficient monitoring of the patient, lack of data on the previous cardiological condition, and lack of serial monitoring of echocardiographic findings. Other challenges, which are related to the aforementioned problem, are the estimation of the variable states of preload and afterload. It is also important to distinguish between patients with systolic and diastolic dysfunction, and base the treatment approach accordingly.

## 3. Pathophysiology

From a pathophysiological point of view, the mechanism of development of myocardial dysfunction in sepsis occurs as a result of direct depression of the myocardium, weakened myocardial circulation, and mitochondrial dysfunction. Direct myocardial depression is a consequence of the pathogen’s direct harmful effects, activation of the host’s immune system itself, with consequent damaging effects primarily of prostaglandins, nitric oxide, and finally apoptosis [4]. Direct depression of the myocardium is a consequence of the reduction of β-adrenergic receptors, which reduces the adrenergic response at the cardiomyocyte level in a process mediated by various pro-inflammatory substances (cytokines and nitric oxide [NO]).

The host’s immune system recognizes the invasion of a pathogenic microorganism by typically identifying pattern recognition receptors (PRRs), which bind to patho-gen-associated molecular patterns (PAMPs). PAMP lipopolysaccharide (LPS), lipo-teichoic acid and others are parts of microorganisms that play a role in conquering the host. On the other hand, as a result of direct cell damage, molecules called damage-associated molecular patterns (DAMPs) are released. DAMPs represent ligands for PRRs that, when activated, promote nuclear translocation of various transcription factors (e.g., nuclear factor kappa-light chain enhancer of activated B cells (NF-κB)) and promote proinflammatory cytokine release [12, 13, 14].

The most common pro-inflammatory cytokines released by macrophages in sepsis are tumor necrosis factor (TNF)-α and interleukin (IL)-1β, *in vitro* they depressant activity of cardiac contractility [15]. NO and free oxygen radicals, which are considered second-degree factors, play a role in myocardial depression during sepsis [16, 17]. The sequence of interconnected mechanisms is as follows: IL-1 is synthesized in response to TNF-α, and IL-1 reduces it by stimulating NO synthase (NOS), i.e., the synthesis of nitric oxide reduces myocardial contractility [18]. The myocardium itself releases IL-6 as a result of the support of α- and β-adrenoreceptors and excessive use of catecholamines. TNF-α alone or in combination with IL-1β is responsible for cardiac depression, which has been proven in *in vitro*, *in vivo*, and also in human studies [19, 20, 21].

Nitric oxide is produced by cardiomyocytes via endothelial nitric oxide synthase (eNOS/NOS3) and has inotropic and relaxing effects in normal hemodynamics. It is also responsible for the metabolism and contractility of cardiomyocytes [22]. Under conditions of increased production or concentration of nitric oxide, it has a negative inotropic effect. Its effects are reflected in changes in calcium concentration within myocardial cells.

A study has found that high levels of endothelin-1 (ET-1) stimulate the release of inflammatory cytokines, which can have adverse effects on the myocardium. Increased expression of intercellular adhesion molecule 1 (ICAM-1) and vascular cell adhesion molecule 1 (VCAM-1) was detected in coronary endothelium and cardiomyocytes [23]. Studies have shown that the molecules have adverse effects on myocardial contractility. *In vivo*, *in vitro*, and human sepsis studies have shown that high histone levels are associated with a higher prevalence of emerging left ventricular dysfunction and new arrhythmias [24]. Circulating histones are also associated with sepsis severity and outcome.

The humoral immune response that is activated in sepsis results in the activation of complement proteins, such as complement C5a. Expression of the C5aR receptor on cardiomyocytes, complement C5a, mediates C5a-induced cardiodepression [25].

Cardiomyocyte apoptosis is a leading cause of sepsis-induced myocardial depression and multiorgan dysfunction. Myocardial dysfunction often progresses with cardiomyocyte apoptosis, after which the number of β-adrenoreceptors decreases, and the functions of myofibrils are damaged due to calcium release disorders [26]. Very low levels of myocyte apoptosis cause life-threatening dilated cardiomyopathy [27]. >30 microRNAs (miRNAs) have been found to be involved in sepsis-induced cardiac dysfunction; among them, >10 miRNAs (such as miR-155, miR-24 and miR-192-5p) are involved in the regulation of sepsis-induced cardiac apoptosis while others inhibit myocyte apoptosis [28, 29].

About 70% of cardiac adenosine triphosphate (ATP) is produced by the oxidation of lipids, the rest is produced by the oxidation of glucose, and a smaller part also comes from the catabolism of lactate and ketone bodies. In sepsis, myocytes use glucose as their primary energy substrate, not fatty acids, which results in a detrimental effect on myocardial contractility, as occurs during post-ischemic hibernation of the myocardium [5].

## 4. The Role of Mitochondrial Dysfunction

Mitochondrial dysfunction is a leading problem in the development of septic cardiomyopathy, it involves oxidative phosphorylation, production of reactive oxygen radicals, reprogramming of energy metabolism and mitophagy. The role of mitochondrial dysfunction is shown by the results of research that compared the hearts of people who died of sepsis, with the hearts of people who undergo transplantation, and confirmed that hearts of patients who died from sepsis showed a significant decrease in the expression of genes related to mitochondrial ATP production [30].

Inflammation and oxidative stress change the structure of mitochondria with consequent development of edema, cytoplasmic accumulation of denatured proteins and development of lysosomal lesions [31, 32]. Such damage disrupts the respiratory chain with a decrease in ATP synthesis, release of calcium and proapoptotic proteins [33]. Previous research has described several mechanisms other than oxidative stress that lead to mitochondrial dysfunction, changes in structure, increased permeability of membranes, mitochondrial separation [34].

Major mechanisms leading to deranged oxidative phosphorylation in septic cardiomyopathy include altered cyclic adenosine monophosphate (cAMP)-dependent protein kinase A signaling, overproduction of reactive oxygen species (ROS) and NO, calcium overload, and reduced antioxidants within mitochondria [35, 36]. The resulting increased production of ROS and NO can lead to direct and indirect damage. Direct damage is oxidative or nitrosative, whereas indirect damage occurs through inhibition of oxidative phosphorylation complexes. Studies have shown that ROS and NO can inhibit mitochondrial complexes I and IV and increase their membrane permeability [37, 38]. Increased mitochondrial inducible nitric oxide synthase (iNOS) activation leads to increased mitochondrial peroxynitrite levels, which has been shown to play an important role in mitochondrial dysfunction during sepsis. Increased mitochondrial uncoupling protein (UCP) expression leads to a decrease in mitochondrial membrane potential and ATP synthesis and also results in proton release, thereby reducing ROS formation [39]. Another proposed mechanism for the development of mitochondrial dysfunction related to oxidative and nitrative stress is the activation of enzymes related to many cellular processes, including DNA repair [40].

Mitochondrial function is maintained through a balance between fission, fusion, biogenesis, and autophagy [41]. Different signaling pathways enable interaction between mitochondria and the nucleus [42]. Mitochondria undergo various morphological changes during fission and fusion, which help maintain a healthy mitochondrial population. It achieves the aforementioned by facilitating the exchange of mitochondrial DNA, preserving the integrity of mitochondrial DNA. Fission and fusion processes are present in stressful conditions and have a key role in eliminating damaged mitochondria [43]. Proteins that play a role in fusion (mitofusin-2) and fission (dynamin-related protein-1) are associated with changes in mitochondrial membrane potentials and reduced oxygen consumption [44]. Mitophagy (autophagic degradation) and mitoptosis (programmed destruction) are processes by which deal with damaged mitochondria.

The most important function of macrophages is phagocytosis of both pathogens and apoptotic cells. Macrophage clearance of innate immune cells may also be impaired by increased IL-10 production from neutrophils and pyroptosis [45].

## 5. Diagnostic Possibilities

Establishing a diagnosis is a major challenge in establishing a diagnosis of septic cardiomyopathy. Due to insufficiently clear and specific criteria, it is difficult to distinguish heart failure from septic cardiomyopathy. Clinical findings suggestive of septic cardiomyopathy are “septic, cold extremities” phenotype, hemodynamic instability despite vasopressor therapy, failure to respond to preload challenge, cardiac arrhythmias, abnormal echocardiogram, low mixed venous oxygen saturation, and elevated cardiac troponins [46].

### 5.1 Echocardiography

In contrast to serum biomarkers, echocardiography provides several possibilities for the diagnosis of septic cardiomyopathy. The method is simple, available, cheap, easily repeatable, and it can be performed “at the bedside” in critically ill patients. Even though at the beginning it was considered that the assessment of reduced LVEF would be sufficient for establishing the diagnosis, the pseudo-normalization of LVEF in the case of reduced preload in distributive shock represents a problem. LVEF is not a sensitive indicator of myocardial contractility but reflects the relationship between LV myocardial contractility and LV afterload. Considering the above, re-evaluation should be done after initial volume replacement and vasopressor administration. Systolic dysfunction of left ventricular is present in 50–60% of patients with sepsis. A retrospective analysis in the intensive care unit, which analyzes the data of echocardiographic findings made within 3 days of admission to the hospital, showed that the largest number of patients had LVEF between 55% and 70%. At the same time, the highest in-hospital mortality was found in patients with LVEF <25%, and those with hyperdynamic myocardium, i.e., LVEF >70% [47].

Echocardiographic tools that can be used to demonstrate septic LV dysfunction include the myocardial performance index (Tei index), which reflects the time spent in isovolumetric contraction, lower values ​​representing better function. Mitral annular plane systolic excursion (MAPSE) can also play an important role in the assessment function LV [48, 49]. Although not many studies have been published so far investigating the role of MAPSE in the assessment of systolic function in septic cardiomyopathy, Brault *et al*. [50] described in their work a positive correlation of septal MAPSE ≤1.2 cm with LV systolic dysfunction. Likewise, MAPSE was shown to be an independent predictor of intra-hospital mortality in pediatric patients with sepsis. Its association with LVEF and troponin I was established in the same population [51]. The leading shortcoming of the mentioned studies is the small sample of patients.

LV diastolic dysfunction is very common in septic shock, and it represents an early biomarker, it has prognostic significance. Based on the 2016 recommendation by the American Society of Echocardiography and the European Association of Cardiovascular Imaging, LV diastolic dysfunction is defined based on left atrium volume assessment (>34 ${\mathrm{mL/m}}^{2}$), tricuspid regurgitation velocity >2.8 m/s, tissue Doppler imaging (TDI) E’ velocity <10 cm/s in the lateral annulus and <7 cm/s in the septal annulus, and E/e’ ratio >13 in the lateral annulus and >15 in the septal annulus [52]. These recommendations enabled the recognition of diastolic dysfunction in 60% of patients with sepsis already on the first day [53]. Meta-analysis of Sanfilippo *et al*. [54] from 2017 showed that the measurement of E’ in the lateral annulus is more strongly associated with prognosis than the measurement in the septal annulus, but this meta-analysis also showed strong association both E’ and mortality in septic patients. Since these are patients who are mechanically ventilated, a meta-analysis that included 11 studies analyzed the influence of ventilation on echocardiographic parameters of systolic and diastolic function. The results of this analysis showed that the failure of weaning from mechanical ventilation is associated with worse diastolic function and increased LV filling pressure [55]. Based on the aforementioned knowledge, Sanfilippo *et al*. [56] proposed a model of the possibility of a therapeutic approach to a critically ill patient with diastolic dysfunction. This model of optimized treatment proposed by the authors is called CHEOPS and includes: ultrasound of the chest (heart and lungs) with hemodynamic assessment depending on heart rate and application of vasoactive support, optimization of mechanical ventilation and hemodynamic stabilization. It should be emphasized that the mentioned model doesn’t initially refer to the resuscitation phase, but to further optimal treatment.

It’s estimated that right ventricular dysfunction is present in 50–55% of cases, while isolated right ventricular dysfunction is present in 47% of cases [57, 58]. Right ventricular dysfunction is defined according: tricuspid annular plane systolic excursion (TAPSE) <16 mm, tricuspid lateral annular systolic velocity (TDI S’ wave) <15 cm/s, and right ventricular fractional area change (FAC) <35% [59]. Lanspa *et al*. [58] have shown in their research that half of patients with sepsis have RV dysfunction, and it is associated with three times higher 28-day mortality. Among the tested parameters of RV dysfunction, TAPSE and FAC stand out.

Strain imaging is a new method based on regional deformation of the myocardium. Global longitudinal strain (GLS) is the most commonly used parameter. Normal GLS for LV is more than –18% and for RV more than –22%. The values of the circumferential stress of the left ventricle at the three levels basal, middle and apex range from –22 to –35% [60]. GLS assessment is certainly desirable in subclinical, early assessment of myocardial damage as part of sepsis, the main disadvantage of such a sophisticated method is the difficult availability in the ICU. Studies have shown that worse GLS (less negative) is associated with higher mortality in patients with sepsis, and the same relationship was not established between mortality and LVEF [61].

### 5.2 Cardiac MRI

MRI of the heart represents a method that is available regardless of the severity of the general condition because it deals with patients who are sedated, mechanically ventilated. Myocardial edema and inflammation are the leading features of septic cardiomyopathy, without the presence of focal fibrosis. Research has shown that in T2 sequences there is homogeneous enhancement of the myocardium, and after the application of gadolinium there was no late thickening of the myocardium [62]. The leading limiting factors for the application of cardiac MRI in daily practice are the duration of the examination in the situation, especially when dealing with hemodynamically unstable patients.

### 5.3 Troponin

In septic patients, elevated cardiac troponin (cTn) correlates with a greater degree of left ventricular dysfunction, disease severity, and mortality. The measurement of cardiac biomarkers in the serum provides separate, but in combination with echocardiographic parameters, has it’s significance in establishing the diagnosis of septic cardiomyopathy [63]. A retrospective analysis showed that elevated levels of troponin T upon admission to the ICU are associated with increased in-hospital mortality, as well as 1-year mortality [64].

### 5.4 NTproBNP (N-Terminal proBrain Natriuremic Peptide)

For NTproBNP, we cannot say for sure that it has a significance in diagnosis, but multicenter clinical studies have found that in patients with sepsis and septic shock, N-terminal pro-B-type natriuretic peptide (NTproBNP) and cTn were elevated in 97.4% and 84.5% of patients, respectively. The association of biomarkers with the development of septic shock and mortality was established [10].

### 5.5 Biomarkers of Mitochondrial Dysfunction

Mitochondrial dysfunction of the heart has been studied in numerous animal models *in vitro* and *in vivo*. Detected mitochondrial changes include altered redox status and reduction of oxygen consumption, ATP generation, changes in mitochondrial membrane potential.

Since we have highlighted in an earlier part of our work the importance of mitochondrial dysfunction in the development of septic cardiomyopathy, it will certainly be important to find biomarkers that can objectify this damage. Blood lactate is the most commonly used marker of mitochondrial dysfunction, but it is not specific enough. Previous studies identified two biomarkers as a result of the integrated mitochondrial response to stress, fibroblast growth factor-21 (FGF-21) and growth differentiation factor-15 (GDF-15) [65]. Circulating GDF-15 is currently the best biomarker for diagnosing mitochondrial dysfunction. Studies have shown that GDF-15 expression can induce stress responses through regulation of activating transcription factor 4 [66]. GDF-15 was found to be significantly increased in skeletal muscle and serum of patients with mitochondrial dysfunction [67]. The study found that the increase in GDF-15 in patients’ serum was associated with the severity of organ damage and sepsis. Dynamic changes in GDF-15 may indicate good diagnostic and prognostic value. GDF-15 is thought to play a protective role in sepsis; it can enhance the phagocytic and bactericidal functions of macrophages [68].

FGF-21 is a growth factor that regulates lipid and glucose metabolism. Earlier studies proved the anti-inflammatory effect of FGF-21 in sepsis, thus explaining the protective mechanism. It maintains thermoregulation and preserves cardiovascular function during bacterial inflammation [69].

Both of the mentioned biomarkers can be easily quantified in serum or plasma using enzyme-linked immunosorbent assays (ELISA). FGF-21 and GDF-15 are new biomarkers, and more sensitive than lactate, which is routinely used. A disadvantage in both cases is that the specificity of these biomarkers for detecting mitochondrial dysfunction in multifactorial diseases has not yet been clarified.

Increasing evidence suggests that mitochondrial dysfunction is also associated with reduced levels of miRNAs [70]. miRNAs serve as regulators of gene expression in biological processes and cell signaling pathways. miRNAs are produced in cells, but extracellular miRNAs can also be present as stable molecules in plasma and body fluids. Due to this property, miRNAs can be used as serum biomarkers of sepsis [28]. Manetti *et al*. [28] their study showed that many miRNAs are involved in cardiac dysfunction caused by atherosclerosis and sepsis. miR-223 and miR-23b stand out, and further studies are needed to determine the clinical significance of these molecules in septic cardiomyopathy.

Prevalence of homoplasmic/heteroplasmic mtDNA (mitochondrial DNA) mutations, SLSMD (single large deletion of mtDNA), MLSMD (multiple large deletion of mtDNA) in blood are probably not very sensitive biomarkers, but can be specific. Identification of mutations in blood can be a strong indicator of mitochondrial damage. Molecular tests for mtDNA can represent an attractive alternative to performing cellular tests [71].

## 6. Therapeutic Possibilities

The main goals of treatment in septic cardiomyopathy are based on the optimization of hemodynamic parameters (fluid replacement, use of inotropes and vasopressors, alternative renal methods, mechanical ventilation) and the use of targeted antibiotic therapy. Treatment should begin with volume supplementation of 20 mL/kg to increase preload and thus cardiac output (CO). Volume replacement in the initial stage is extremely important, as later complications of pulmonary edema may occur due to increased pulmonary microcirculatory permeability and vasodilation [62]. Current international guidelines for sepsis treatment recommend “hemodynamic monitoring” [72]. In this setting, echocardiography plays a key role, helping to differentiate between hypovolemic patients and volume overloaded patients. Norepinephrine, an alpha and beta agonist, is the vasopressor of choice in patients with sepsis and can cause hypotension despite adequate volume compensation [73]. Current guidelines support the use of dobutamine in the presence of myocardial dysfunction, such as elevated filling pressures and low cardiac output or signs of sustained hypoperfusion [71]. Taking catecholamines increases the risk of developing cardiac arrhythmias. Due to its mechanism of action, levosimendan has a more favorable inotropic effect that is independent of β-adrenergic activity and selectively binds to calcium-saturated troponin C, thereby increasing myocardial contractility [74]. Levosimendan does not cause the accumulation of calcium in cardiomyocytes, but increases sensitivity to existing calcium, thereby reducing oxygen consumption and the occurrence of ischemia, without developing tolerance. Liu *et al*. [75] showed in their meta-analysis that the use of levosimendan had no effect on mortality or LVEF but had a more favorable effect on myocardial dysfunction in patients with sepsis compared with the use of dobutamine. The adrenergic system plays an important role in sepsis, and β-adrenergic modulation should be considered as a therapeutic intervention. Short-acting beta-blockers are preferred when there is a risk of hemodynamic instability. They have limited effects on blood pressure, have positive inotropic effects, and are highly effective in reducing heart rate [76]. A meta-analysis that included 7 randomized controlled trials (RCTs) showed that the use of ultrashort-acting β-blockers, esmolol and landiolol in patients with sepsis and persistent tachycardia is associated with reduced 28-day mortality [77]. In contrast to that, a recently published study, which examined the effect of landiolol administration in patients with sepsis and tachycardia along with the administration of norepinephrine, didn’t show a favorable effect on the SOFA (Sequential Organ Failure Assessment) score, as well as on 28-day mortality [78]. Cardiogenic shock caused by sepsis, if it doesn’t respond to applied conservative treatment, is considered indication for veno-arterial (VA) extracorporeal membrane oxygenation (ECMO), which can serve as a bridging method, and it can be associated with a better prognosis [79]. VA-ECMO increases afterload and decreases SV, and ensures systemic perfusion.

## 7. Conclusions

Myocardial dysfunction is only one of the organ dysfunctions that can be present in patients with sepsis, and its presence is associated with a worse prognosis. In this review, it was shown that the mechanism of development of septic cardiomyopathy is complex and includes a series of procedures that will lead to cardiomyocyte damage. In particular, we developed mitochondrial dysfunction. Mitochondrial dysfunction is one of the key mechanisms in the development of septic cardiomyopathy. Current knowledge suggests that GDF-15 and FGF-21 would be good markers of mitochondrial dysfunction. Advances in diagnostic methods, primarily echocardiography, have made it possible to detect abnormalities, but the methods are not specific enough, and because of the above, future research should focus on biomarkers that, in addition to available diagnostic methods, will enable early recognition and targeted treatment. When we talk about echocardiography, as we described in the paper, there are a number of modalities that can detect dysfunction of the myocardium, left or right ventricle, but it is certainly necessary to find additional biomarkers that, in combination with existing imaging methods, will enable a simpler assessment and final diagnosis. Previous studies have confirmed that the assessment of LV diastolic dysfunction correlates better with the prognosis and mortality of patients with sepsis compared to LVEF. Among the other methods, it is necessary to single out GLS, which proved to be a good predictor of subclinical signs of myocardial dysfunction, but the main drawback is the difficult availability in the ICU. Previous research has been conducted primarily on animal models, so certainly research in real clinical practice will provide a new perspective in the diagnosis and treatment of cardiomyopathy caused by sepsis. Septic cardiomyopathy will represent a challenge to many researchers in the future, both in the diagnostic and the therapeutic approach.
